# All-*Trans* Retinoic Acid Modulates DNA Damage Response and the Expression of the *VEGF-A* and *MKI67* Genes in ARPE-19 Cells Subjected to Oxidative Stress

**DOI:** 10.3390/ijms17060898

**Published:** 2016-06-14

**Authors:** Paulina Tokarz, Agnieszka Wanda Piastowska-Ciesielska, Kai Kaarniranta, Janusz Blasiak

**Affiliations:** 1 Department of Molecular Genetics, University of Lodz, 90-236 Lodz, Poland; ptokarz@biol.uni.lodz.pl; 2 Department of Comparative Endocrinology, Medical University of Lodz, 90-752 Lodz, Poland; agnieszka.piastowska@umed.lodz.pl; 3 Department of Ophthalmology, University of Eastern Finland, 70210 Kuopio, Finland; kai.kaarniranta@kuh.fi; 4 Department of Ophthalmology, Kuopio University Hospital, 70210 Kuopio, Finland

**Keywords:** AMD, ARPE-19 cells, ATRA, cell death, DDR, DNA damage response, oxidative stress, retinoic acid, ROS

## Abstract

Age-related macular degeneration (AMD) is characterized by the progressive degradation of photoreceptors and retinal pigment epithelium (RPE) cells. ARPE-19 is an RPE cell line established as an *in vitro* model for the study of AMD pathogenesis. Oxidative stress is an AMD pathogenesis factor that induces DNA damage. Thus, the oxidative stress-mediated DNA damage response (DDR) of ARPE-19 cells can be important in AMD pathogenesis. The metabolism of retinoids—which regulates cell proliferation, differentiation, and the visual cycle in the retina—was reported to be disturbed in AMD patients. In the present work, we studied the effect of all-*trans* retinoic acid (ATRA, a retinoid) on DDR in ARPE-19 cells subjected to oxidative stress. We observed that ATRA increased the level of reactive oxygen species (ROS), alkali-labile sites in DNA, DNA single-strand breaks, and cell death evoked by oxidative stress. ATRA did not modulate DNA repair or the distribution of cells in cell cycle in the response of ARPE-19 cells to oxidative stress. ATRA induced autophagy in the absence of oxidative stress, but had no effect on this process in the stress. ATRA induced over-expression of proliferation marker *MKI67* and neovascularization marker *VEGF-A*. In conclusion, ATRA increased oxidative stress in ARPE-19 cells, resulting in more lesions to their DNA and cell death. Moreover, ATRA can modulate some properties of these cells, including neovascularization, which is associated with the exudative form of AMD. Therefore, ATRA can be important in the prevention, diagnosis, and therapy of AMD.

## 1. Introduction

Age-related macular degeneration (AMD) is a disease in which specialized neurons (photoreceptors) undergo cell death in the central part of the retina—the macula—leading to the complete loss of central vision. The photoreceptor degeneration in AMD is a consequence of the dysfunction of the retinal pigment epithelium (RPE) located between photoreceptors and the vascular layer that provides photoreceptors with oxygen and nutrients and removes waste material [1]. RPE dysfunction results from the accumulation of lipofuscin—granules containing deposits of non-completely digested material produced by photoreceptors—in RPE. Typically, AMD is classified into two subgroups, (I) atrophic (dry/non-exudative) AMD, in which cells degenerate in the macular area; and (II) exudative (wet/neovascular) AMD, resulting from choroidal neovascularization mediated by vascular endothelial growth factor A (VEGF-A) [2].

Oxidative stress in the retina results from high consumption of oxygen, exposure to blue and UV lights and the phagocytic functions of RPE cells [3,4,5,6]. Thus, RPE cells are particularly exposed to reactive oxygen species (ROS), and indeed ROS-induced damage is frequently observed in AMD patients [7]. ROS can damage DNA/RNA, lipids, proteins, and other macromolecules. Oxidative stress occurs in the retina before the onset of AMD, and it can participate in AMD induction and progression. An excess of ROS in the retina can be inactivated by antioxidant enzymes or low molecular weight antioxidants. The application of the antioxidant cocktail known as the AREDS formulation led to a delay in the progression of AMD, but it did not inhibit AMD completely [8]. Impairments in the cellular antioxidant system, including lower activity of sodium dismutase (SOD) and glutathione peroxidase (GPx)—which were observed in AMD—can lead to ROS-induced DNA damage [9]. We previously showed that AMD patients accumulated DNA damage, including oxidative modification of DNA bases, and were more sensitive to DNA-damaging agents [7,10]. Aside from antioxidant enzymes and low molecular weight antioxidants (which neutralize ROS) the DNA damage response (DDR) can overcome the consequences of oxidative stress-induced DNA lesions. DDR is a complex, multi-pathways reaction including chromatin remodeling, DNA repair, cell cycle regulation, autophagy, and apoptosis. We showed that lymphocytes from AMD patients repaired DNA lesions less efficiently than controls [7,10]. Others showed that post-mortem AMD retinas had a significantly higher number of apoptotic cells than normal retinas, indicating that DDR can be impaired in AMD [11].

Retinoids in the retina are involved in transcriptional regulation and the visual cycle [12]. Defects in retinoid metabolism are associated with the accumulation of cytotoxic and ROS-generating lipofuscin in RPE [13]. The inhibition of the visual cycle decreased the accumulation of lipofuscin and rendered cells more resistant to light-induced damage [14]. A synthetic retinoid derivative—fenretinide, which decreases the level of substrates for the visual cycle—has already been evaluated in the treatment of dry AMD in the clinical trial phase II [15].

All-*trans* retinoic acid (ATRA), a natural vitamin A derivative, induces cell differentiation that arrests cells in the G0/G1 phase. ATRA binds to the retinoic acid receptor (RAR), which, together with activated retinoid X receptor (RXR), binds to retinoic acid response elements (RAREs) in DNA. This interaction stimulates the down-regulation of DNA methyltransferases (DNMT)—a gene expression co-activator [16]. ATRA was reported to modulate the expression of many DDR proteins, including ATM, TP53, Bcl-2 and caspases, suggesting that ATRA can modulate DDR [17,18].

In the present study, we characterized the modulation of DDR pathways by ATRA in ARPE-19 cells. We pre-treated ARPE-19 cells with ATRA at therapeutic concentrations and exposed them to oxidative stress and quantified the level of intracellular ROS, DNA damage, phosphorylation of ATM (ataxia teleangiectasia mutated) and H2AX histone variant, cell death and autophagy, the kinetics of DNA damage repair, and the distribution of cells in the cell cycle.

## 2. Results

### 2.1. All-Trans Retinoic Acid (ATRA) Increases the Level of Intracellular Reactive Oxygen Species and Oxidative Stress-Induced DNA Damage

**Figure 1:**
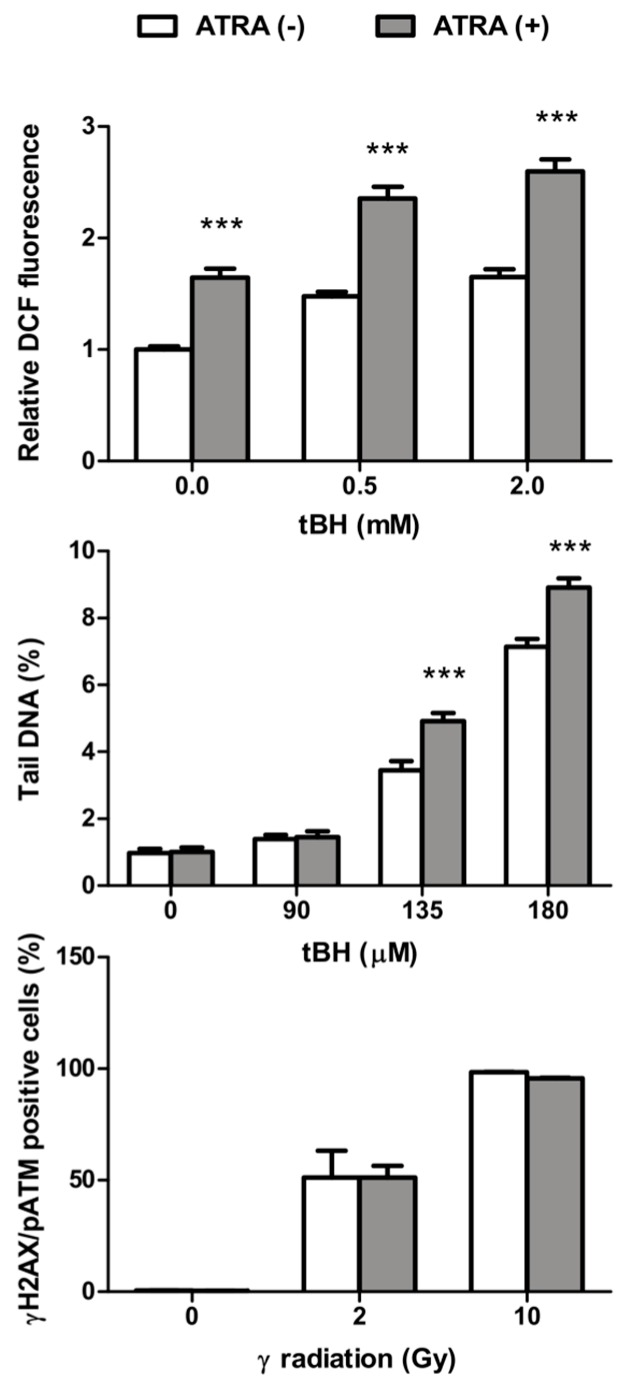
Mean intracellular reactive oxygen species (ROS) levels and DNA damage in all-*trans* retinoic acid (ATRA)-treated and control ARPE-19 cells exposed to *tert*-butyl hydroperoxide (tBH). Reactive oxygen species (ROS) are expressed as the relative fluorescence of 2′,7′-dichlorofluorescein (DCF) normalized to controls after 30 min incubation with tBH at 37 °C. DNA damage was measured as a percentage of tail DNA in the comets in alkaline versions of the comet assay following exposure to tBH for 15 min at 37 °C. The induction of DNA double strand breaks was assessed by the extent of phosphorylation of H2AX and ATM (ataxia teleangiectasia mutated) (γH2AX and pATM) after γ-ray irradiation (2 Gy/min) of cells. Results are presented as mean ± SEM, *n* = 12 for ROS level, *n* = 100 for comet assay, *n* = 8 for H2AX/ATM phosphorylation, *** *p* < 0.001.

Since ARPE-19 cells were treated with an oxidant, we checked whether ATRA could modulate the level of intracellular ROS in oxidative stress. The dichlorofluorescein (DCF) assay was used to detect ROS in ATRA-treated and control ARPE-19 cells after exposure to *tert*-butyl hydroperoxide (tBH). Results showed that ROS generation after tBH exposure gradually increased in both ATRA-treated and control ARPE-19 cells (Figure 1). ROS production in ATRA-treated cells was significantly higher than in the control group in normal conditions and under oxidative stress.

As oxidative stress can induce DNA damage, we checked whether it could be modulated by ATRA. Figure 1 shows DNA damage in ATRA-treated and control ARPE-19 cells in the alkaline version of the comet assay after tBH treatment. This version of the assay is sensitive towards DNA single-(SSBs) and double-strand breaks (DSBs) and alkali-labile sites. ATRA alone did not induce DNA damage in ARPE-19 cells. tBH increased DNA damage in a concentration-dependent manner in ATRA-treated and control ARPE-19 cells. The extent of tBH-induced DNA damage in ATRA-modulated ARPE-19 cells was more pronounced than in control.

Additionally, we performed the immunostaining of γH2AX and phosphorylated ATM, markers of DSBs, after exposure of ATRA-treated and control ARPE-19 cells to γ-ray radiation. After γ-ray exposure, phosphorylation of H2AX and ATM gradually increased in ATRA-treated and control cells (Figure 1). ATRA did not introduce DSBs to ARPE-19 cells. There was no difference between the activation of H2AX and ATM in ATRA-treated and control cells following γ-ray irradiation.

### 2.2. ATRA Potentiates Cell Death Induced by Oxidative Stress

**Figure 2:**
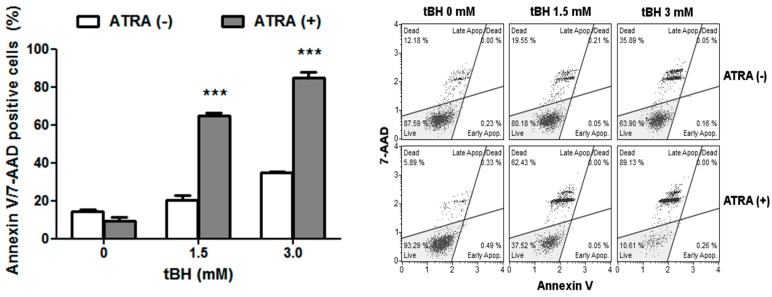
Cell death of ATRA-treated and control ARPE-19 cells exposed to *tert*-butyl hydroperoxide (tBH) for 4 h at 37 °C as assessed by flow cytometry with Annexin V/7-AAD staining. Results are presented as mean ± SEM, *n* = 8, *** *p* < 0.001. Representative fluorescence activated cell sorting (FACS) analysis of Annexin V labeling profiles are presented.

The cell death of the ATRA-treated and control ARPE-19 cells incubated with tBH was assessed using the Annexin V/7-AAD assay. The results (Figure 2) show that after tBH treatment for 4 h, the number of viable cells decreased and the proportion of dead cells increased in a dose-dependent manner for each group of cells. ATRA did not evoke cell death in ARPE-19 cells after 72 h incubation. The fraction of dead cells was significantly higher in ATRA-treated than control ARPE-19 following tBH treatment. We can speculate that there were mainly necrotic cells, as early apoptotic cells were hardly observed.

### 2.3. ATRA Does Not Change DNA Repair

**Figure 3:**
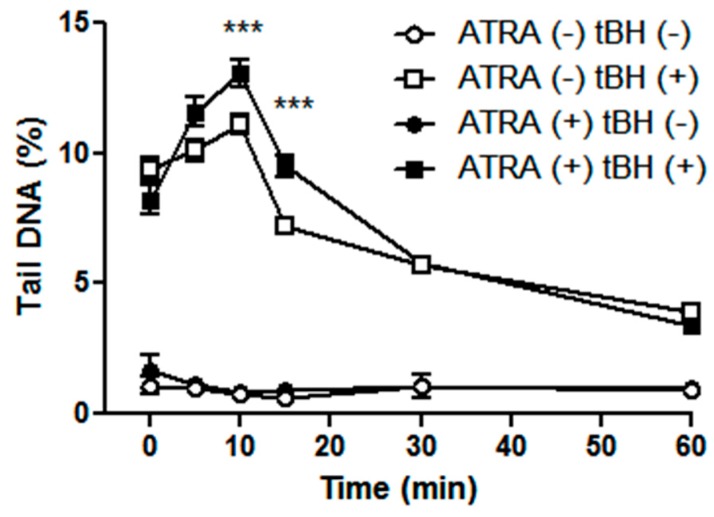
Time course of DNA repair in ATRA-treated and control ARPE-19 cells exposed to 180 μM *tert*-butyl hydroperoxide (tBH) for 15 min at 37 °C. After the exposure, the oxidant was washed out and the cells were incubated in medium at 37 °C for indicated time points. DNA damage was assessed as percentage of tail DNA in the comet in the alkaline version of the comet assay. Results are presented as mean ± SEM, *n* = 100, *** *p* < 0.001.

We analyzed the kinetics of DNA damage repair in ATRA-treated and control ARPE-19 cells in oxidant-free conditions after the generation of DNA damage by tBH exposure. DNA damage immediately after exposure, as well as 5, 10, 15, 30, and 60 min thereafter, was measured as the percentage of tail DNA. In addition, DNA damage in ATRA-treated and control ARPE-19 cells without oxidant was measured. Despite the statistical significance of the difference between DNA damage at two different time points of DNA repair in ATRA-treated and control cells (11.1% *vs.* 13.1% and 7.2% *vs.* 9.2%), DNA damage repair curves were nearly identical and thus we consider this difference as biologically irrelevant (Figure 3). We conclude that ATRA has no influence on DNA damage repair kinetics in ARPE-19 cells.

### 2.4. ATRA Induces Autophagy in Normal Conditions and Does Not Influence this Process in Oxidative Stress

**Figure 4:**
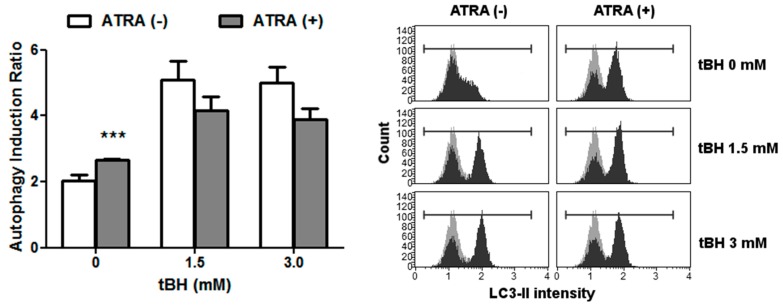
Autophagy of ATRA-treated and control ARPE-19 cells exposed to *tert*-butyl hydroperoxide (tBH) for 4 h at 37 °C as assessed by flow cytometry analysis of LC3-II staining. Results are presented as mean ± SEM, *n* = 8, *** *p* < 0.001. Representative FACS analysis of LC3-II labeling profiles are presented.

Emerging evidence suggests the involvement of autophagy in DDR, so we decided to check the modulation of this process by ATRA. Since conversion of LC3-I to LC3-II correlates well with the number of autophagosomes, the amount of LC3-II was used as an indicator of autophagic activity (Figure 4). Up-regulation of LC3-II following tBH treatment was observed for ATRA-treated and control ARPE-19 (*p* < 0.001). ATRA induced accumulation of LC3-II in ARPE-19 in normal conditions, but not under oxidative stress.

### 2.5. ATRA Does Not Influence Cell Cycle Regulation in Oxidative Stress

**Figure 5:**
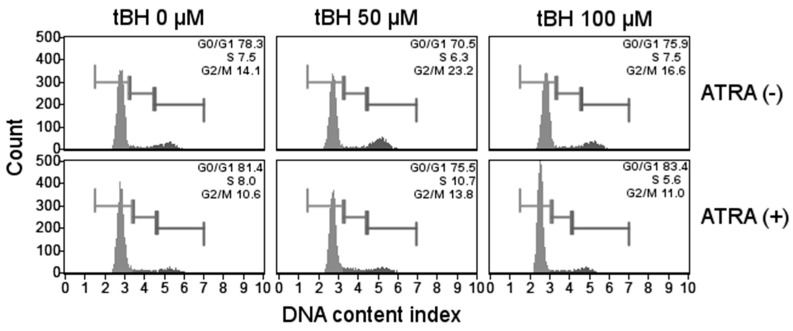

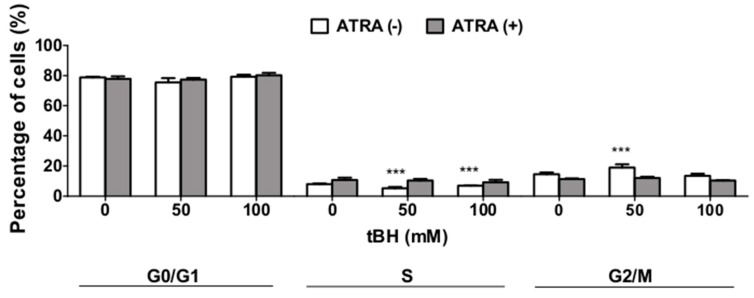
Cell cycle of ATRA-treated and control ARPE-19 cells exposed to *tert*-butyl hydroperoxide (tBH) for 24 h at 37 °C as assessed by flow cytometry analysis of propidium iodide (PI) staining. Results are presented as mean ± SEM, *n* = 8, *** *p* < 0.001. Representative FACS analysis of cell cycle profiles are presented.

In order to examine the effect of tBH on the cell cycle of ATRA-treated and control cells, we determined the DNA content by flow cytometry and propidium iodide (PI) staining. As shown in Figure 5, incubation with increasing concentrations of tBH did not evoke any change in the proportion of ATRA-treated and control ARPE-19 cells in the G0/G1 phase. Dose-response analysis showed that ATRA-treated ARPE-19 cells decreased the proportion of cells in the G2/M, which was accompanied by a slight increase in the S phase when compared to proliferating cells. This may indicate that some ATRA-treated cycling cells were preferentially-delayed in intra-S-phase, although it accounted for 5%–6% of the population only. Thus, we consider this biologically irrelevant.

### 2.6. ATRA Increases the Expression of VEGF-A and MKI67

**Figure 6:**
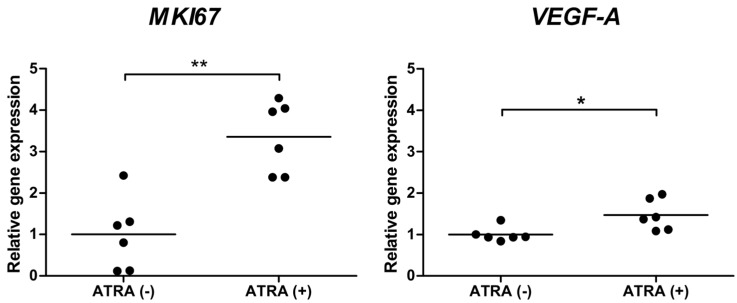
Gene expression of *VEGF-A* and *MKI67* in ATRA-treated and control ARPE-19. The analysis was conducted using Quantitative Real Time-PCR. *GAPDH* was used as a reference gene and the expression of target genes in the control group was assigned a value of 1.0. Displayed is the mean ± SEM, *n* = 6, * *p* < 0.05, ** *p* < 0.01.

Since ATRA can promote RPE differentiation and angiogenesis, we investigated whether ATRA regulates the expression of neovascularization (*VEGF-A*) and G0 state (*MKI67*) markers in ARPE-19 cells. We found that ATRA increased the mRNA expression of both *VEGF-A* and *MKI67* in ARPE-19 cells (Figure 6).

## 3. Discussion

We studied the effect of ATRA on DDR in ARPE-19 cells in oxidative stress, an AMD pathogenesis factor. To study ROS, DNA damage and repair, and cell cycle, we induced oxidative stress with tBH at a concentration which did not affect cell viability. Higher concentrations of tBH were used to assess apoptosis and autophagic cell death. This followed from the different sensitivity of these processes to oxidative stress. We found that ATRA sensitized ARPE-19 cell to oxidative stress by increasing intracellular ROS, DNA damage, and cell death. Autophagy and the expression of the *VEGF-A* gene—a marker of neovascularization and *MKI67*, a marker of G0 cells—increased in ARPE-19 cells upon ATRA treatment.

We observed that 1 µM ATRA was not cytotoxic for ARPE-19 cells as previously shown [19,20,21,22,23], and promoted the generation of intracellular ROS, DNA damage, and apoptosis/necrosis induced by oxidative stress. To analyze intracellular ROS, we applied the commonly used the DCF assay, which does not discriminate between various ROS species. In line with the increased intracellular ROS, we observed the increase in DNA damage assessed as the percentage of tail DNA in comet assay. This technique allows for the quantification of DNA breaks and alkali-labile sites in DNA in a single cell. Together with the visualization of DNA damage by comet assay, we studied the phosphorylation of ATM/H2AX—markers of DSBs. We did not note any difference in the phosphorylation of ATM/H2AX between ATRA-treated and non-treated cells following oxidative stress. Previous studies showed that ATRA up-regulated the expression of ATM, which participated in cell differentiation [17]. Our results suggest that ATRA could potentiate the induction of alkali-labile sites and/or SSBs, but not DSBs.

Cells protect themselves from oxidative damage by low molecular weight antioxidants, antioxidant enzymes, and DNA repair. We evaluated the role of DNA repair in the ATRA-mediated sensitization of ARPE-19 cells to oxidative stress and found that ATRA did not globally influence DNA repair. Thus, we propose that ATRA may exert its biological effect through a mechanism independent of DNA repair, such as the down-regulation of antioxidant enzymes [24]. However, further studies on other DNA repair pathways are necessary to support the conclusion that ATRA does not influence DNA repair.

Together with the increase in intracellular ROS and DNA damage, we observed the increase of cell death when ATRA pre-treated ARPE-19 cells were challenged with oxidative stress. Our observation is in accordance with previous studies, showing that ATRA reduced the expression of Bcl-2, an anti-apoptotic protein, and increased the expression of caspases [25]. ATRA was shown to exert its pro-apoptotic effect through the activation of the mitochondrial pathway [26]. The induction of apoptosis was associated with DNA fragmentation and cell cycle redistribution [26,27].

Together with an increase in cell death, we observed the activation of autophagy in response to oxidative stress, but ATRA had no effect on this process. However, it induced autophagy in normal conditions. Our results contradict those showing no LC3-II staining or faster turnover of LC3-II in oxidant-treated ARPE-19 cells; however, we used a 10× higher concentration of tBH, which may account for this discrepancy [28]. In our study, we stained cells for the presence of LC3-II. ATRA was demonstrated to prevent the accumulation of LC3-II due to faster turnover of the LC3-II protein; thus, we cannot exclude the involvement of autophagy in the ATRA-mediated sensitization of ARPE-19 cells to oxidative stress [29,30,31]. Further research with different autophagic markers should be conducted to study this aspect.

Although ATRA was shown to modulate the expression of TP53 in different cell lines [16,17,32,33], we did not observe any change in the distribution of cells in cell cycle, which suggests normal activation of cell cycle checkpoints. Lack of the ATRA-mediated effect on cell cycle is in agreement with unaffected DNA repair. We applied PI staining to analyze the cell cycle, but this method does not distinguish between the cells in G0 and G1 phases. Thus, we studied the expression of proliferation marker *MKI67* and found that it increased upon ATRA pre-treatment. Therefore, although ATRA did not modulate the cell cycle checkpoints, it could cause the cells to enter G0 phase. ATRA is a potent regulator of cell proliferation that promotes cell differentiation (which is associated with cell cycle exit) in different cell types, and increased the expression of the differentiation markers RPE65 and CRALBP in RPE cells [34,35,36,37].

We observed that the expression of *VEGF-A*, a marker of neovascularization, was up-regulated upon ATRA pre-treatment. A similar result was obtained by others who showed that ATRA increased the expression of *VEGF-A* mRNA and protein in a dose- and time-dependent manner in ARPE-19 cells [37]. The over-expression of *VEGF-A* in the retina influences cells in the choriocapillaries and stimulates subretinal neovascularization—hallmarks of AMD. Currently, the only pharmacological treatment of AMD is the application of *VEGF-A* blocker—either lucentis/ranibizumabum or avastin/bevacizumab—emphasizing the necessity to understand the molecular cause of *VEGF-A* over-expression observed in AMD. The studies on the ATRA-mediated transcriptional regulation of *MKI67* and *VEGF-A* should be extended by proteomic analysis, including the level of expression and activity of the Ki67 and VEGF proteins. The activation of VEGF is strongly associated with the activation of Nrf2—A transcription factor which enhances the expression of cytoprotective genes through the antioxidant response element (ARE) enhancer [38]. In human cells derived from liver hepatocellular carcinoma, embryonic kidney, mammary adenocarcinoma, and others, ATRA acted as an inhibitor of Nrf2 [39,40]. We hypothesize that the sensitization of ARPE-19 cells toward oxidative stress can be partially mediated by the reduction of the Nrf2-driven expression of antioxidant genes.

In conclusion, ATRA sensitized ARPE-19 cells to oxidative stress-induced cell death. This process may be mediated in part by ATRA-associated increase in intracellular ROS production and DNA damage. ATRA exerted its effect neither through modulation of DNA repair nor activation of cell cycle checkpoints. Due to synergistic cytotoxic effects of ATRA and oxidative stress, we suggest the possibility of new therapeutic strategies for AMD based on the inhibition of retinoic metabolism pathways.

## 4. Materials and Methods

### 4.1. Cell lines, Viability, and Treatment

ARPE-19, a human RPE cell line, was purchased in the American Type Culture Collection (ATCC, Manassas, VA, USA). The cells were cultivated in DMEM:F-12 1:1 mixture medium with 15 mM HEPES (Lozna, Basel, Switzerland) containing 10% inactivated fetal bovine serum (FBS) (Biowest, Nuaillé, France), 2 mM l-glutamine (Lonza), 100 units/mL penicillin (Lonza), and 100 μg/mL streptomycin (Lonza). The cells were cultured in a 5% CO_2_ humidified incubator at 37 °C and treated with 1 µM all-*trans* retinoic acid (ATRA) (Sigma, Munich, Germany) for 72 h and then exposed to *tert*-butyl hydroperoxide (tBH) or γ radiation with a dose rate of 2 Gy/min. Cell viability was assessed by trypan blue exclusion assay in at least 100 cells.

### 4.2. Intracellular Reactive Oxygen Species

Following the treatment with ATRA, the cells were washed twice with Hank’s balanced salt solution (HBSS) containing Ca^2+^ and Mg^2+^ and stained with 5 µM 2′,7′-dichlorodihydrofluorescein diacetate (H_2_DCF-DA) (Life Technologies, Budapest, Hungary) in HBSS containing Ca^2+^, Mg^2+^ (Lonza) for 30 min in the dark. Then, the cells were washed twice and incubated with tBH. After incubation with tBH, the cells were washed twice and fluorescence intensity was measured with λ_ex_ = 485/20 nm and λ_em_ = 528/20 nm using a Synergy HT spectrophotometer (Biotek, Bad Friedrichshall, Germany).

### 4.3. DNA Damage—The Comet Assay

We studied the induction of DNA damage using comet assay in the alkaline condition (pH > 13) [41,42,43]. Briefly, the cells were seeded at a density of 5 × 10^4^ cell/mL in medium in 12-well culture plates and treated as described above. Then, the cells were collected, centrifuged, and prepared as previously described [44]. From one hundred comets, the percentage of tail DNA was scored from each sample.

### 4.4. DNA Damage—Phosphorylation of H2AX and ATM

After the treatment, the cells were washed with phosphate buffered saline (PBS), resuspended in fresh medium, and incubated for 1 h at 37 °C to observe H2AX and ATM phosphorylation, as described previously [45]. The activation of ATM and H2AX was determined using the Muse^TM^ Multi-Color DNA Damage kit (Millipore, Hayward, CA, USA) according to the manufacturer's instructions. Following the treatments, the cells were washed with PBS, resuspended in 1× Assay Buffer, mixed with Fixation Buffer (1:1), and allowed to cool. Then, the cells were permeabilized with ice-cold 1× Permeabilization Buffer, resuspended in 1× Assay Buffer, and stained with anti-phospho-ATM (Ser1981)-PE and anti-phospho-Histone H2AX (Ser139)-PECy5 conjugated antibodies for 30 min at room temperature in the dark. The excess of dyes was washed out with ice cold 1× Assay Buffer, and the samples were quantified by Muse Cell Analyzer (Millipore, Hayward, CA, USA). The events for ATM activated cells, H2AX activated cells, cells with DNA double-strand breaks determined as dual activation of both ATM and H2AX, and negative cells, were counted with the Muse Cell Analyzer (Millipore, Hayward, CA, USA) and analyzed with MuseSoft 1.4.0.0 (Millipore, Hayward, CA, USA).

### 4.5. DNA Repair

Following the treatment with tBH, the oxidant was washed out with PBS and the cells were resuspended in a fresh medium containing 10% FBS. The cells were allowed to repair for 5, 10, 15, 30 or 60 min. Then, the cells were collected and comet assay was performed.

### 4.6. Cell Death

For measurement of cell death, the Annexin V & Dead Cell kit was used (Millipore, Hayward, CA, USA) according to the manufacturer's instructions. Briefly, after treatment, the cells were incubated with Annexin V and Dead Cell Reagent (7-AAD) for 20 min at room temperature in the dark, and the events for dead, late apoptotic, early apoptotic, and live cells were counted with the Muse Cell Analyzer (Millipore, Hayward, CA, USA) and analyzed with MuseSoft 1.4.0.0 (Millipore).

### 4.7. Autophagy

For the analysis of autophagic vacuoles, the cells after treatment were washed and stained using the Autophagy LC3-antibody-based kit (Millipore, Hayward, CA, USA) according to the manufacturer’s instructions. Briefly, the cells were incubated with Autophagy Reagent A in Earle’s balanced salt solution (EBSS) for 5 h at 37 °C to protect the autophagosome-associated LC3 (LC3-II) protein against degradation while washing away cytosolic LC3 (LC3-I). Then, the cells were washed with ice cold HBSS and stained with anti-LC3 Alexa Fluor^®^555 in 1× Autophagy Reagent B on ice for 30 min in the dark. Next, the excess of dye was washed out with ice cold 1× Assay Buffer and the samples were quantified by flow cytometry in Muse Cell Analyzer (Millipore, Hayward, CA, USA). Autophagy Induction Ratio (test sample fluorescence relative to control) was analyzed with MuseSoft 1.4.0.0.

### 4.8. Cell Cycle Analysis

The analysis of the cell cycle distribution was performed using the Muse Cell Cycle kit according to the manufacturer’s instructions. Briefly, after the 24 h treatment with tBH, the cells were collected, washed twice with PBS, and resuspended in PBS to a concentration 1 × 10^6^ cell/mL. The cells were incubated on ice for 15 min, and then while vortexing the samples, one volume of −20 °C absolute ethanol was added dropwise. The samples were stored at 4 °C until analysis, when the cells were pelleted (300× *g*, 20 min) and incubated in Muse Cell Cycle Test Reagent for 30 min at room temperature in the dark. After staining, the cells were analysed with Muse Cell Analyzer. Percentage of cells in G0/G1, S, and G2/M phases were determined using the MuseSoft 1.4.0.0 (Millipore).

### 4.9. Gene Expression

After treatment, total RNA was isolated using ExtractMe Total RNA Kit (Blirt S.A., Gdańsk, Poland) according to manufacturer’s protocol. Quantity and quality of the isolated RNA was assessed through the measurement on the Synergy HT spectrofluorimeter (BioTek Instruments, Winooski, VT, USA). The quantitative real-time PCR reaction was carried out using 2× SensiFAST Probe No-ROX One-Step (Bioline Reagents, London, UK) kit. The analysis of the expression of target genes (*MKI67* and *VEGFA*) was performed using TaqMan^®^ Gene Expression Assay (Life Technologies, Foster City, CA, USA) according to manufacturer's instructions. The *GAPDH* (glyceraldehyde 3-phosphate dehydrogenase) gene was used as a reference. The assay numbers for these genes were as follows: Hs01032443_m1 for *MKI67*, Hs00900055_m1 for *VEGF-A*, and Hs_99999905_m1 for *GAPDH*. Each PCR reaction was performed in 10 μL volume that included 5 μL of 2× SensiFAST Probe No-ROX One-Step Mix (Bioline Reagents, London, UK), 0.1 μL of Reverse Transcriptase (Bioline Reagents, London, UK), 0.2 μL of RiboSafe RNAse Inhibitor (Bioline Reagents, London, UK), 4.2 μL of water diluted cDNA template (100 ng), 0.5 μL of TaqMan^®^ Gene Expression Assay. The quantitative Real-Time PCR reaction was carried out using the CFX96 Touch^TM^ Real-Time PCR Detection System (Bio-Rad, Hercules, CA, USA) in the following conditions: reverse transcription for 20 min at 45 °C, denaturation for 2 min at 95 °C, followed by 40 cycles of 5 s at 95 °C, 1 min annealing and extension at 60 °C. Relative RNA quantification was performed using the 2^−ΔΔ*C*t^ method [46].

### 4.10. Statistical Analysis

Statistical analyses were conducted using GraphPad Prism 5 Software (GraphPad Software, Inc., La Jolla, CA, USA). Comparisons between cells with and without ATRA treatment were performed using Mann–Whitney *U* test. Data were expressed as mean ± SEM.

## 5. Conclusions

Although the prevalence of AMD increases, there is not a significant progress in the treatment of this disease, especially its dry form. Fenretinide, a synthetic retinoid derivative is under clinical trial for dry AMD. In the present work we showed that another retinoid, ATRA, sensitized ARPE-19 cells to oxidative stress-induced cell death, which was associated with increase in intracellular ROS production and DNA damage, without changes in DNA repair and with no activation of cell cycle checkpoints. Since ATRA alone also increased the expression of *VEGF-A*, we conclude that the inhibition of retinoic metabolism pathways is a good candidate for new treatments of dry AMD.

## Abbreviations

ATRA	All-*trans* retinoic acid
AMD	Age-related macular degeneration
DSB	DNA double-strand break
DCF	Dichlorofluorescein
DDR	DNA damage response
FACS	Fluorescence activated cell sorting
PI	Propidium iodide
RPE	Retinal pigment epithelium
ROS	Reactive oxygen species
SSB	DNA single-strand break
tBH	*Tert*-butyl hydroperoxide
